# Does Visceral Osteopathic Treatment Accelerate Meconium Passage in Very Low Birth Weight Infants?- A Prospective Randomized Controlled Trial

**DOI:** 10.1371/journal.pone.0123530

**Published:** 2015-04-15

**Authors:** Nadja Haiden, Birgit Pimpel, Alexandra Kreissl, Bernd Jilma, Angelika Berger

**Affiliations:** 1 Department of Pediatrics, Division of Neonatology, Pediatric Intensive Care Medicine and Neuropediatrics, Medical University of Vienna, Vienna, Austria; 2 Department of Clinical Pharmacology, Medical University of Vienna, Vienna, Austria; Cardiff University, UNITED KINGDOM

## Abstract

**Background:**

To determine whether the complementary approach of visceral manipulative osteopathic treatment accelerates complete meconium excretion and improves feeding tolerance in very low birth weight infants.

**Methods:**

This study was a prospective, randomized, controlled trial in premature infants with a birth weight <1500 g and a gestational age <32 weeks who received a visceral osteopathic treatment 3 times during their first week of life or no treatment.

**Results:**

Passage of the last meconium occurred after a median of 7.5 days (95% confidence interval: 6–9 days, n = 21) in the intervention group and after 6 days (95% confidence interval: 5-9 days, n = 20,) in the control group (p = 0.11). However, osteopathic treatment was associated with a 8 day longer time to full enteral feedings (p = 0.02), and a 34 day longer hospital stay (Median = 66 vs. 100 days i.e.; p=0.14). Osteopathic treatment was tolerated well and no adverse events were observed.

**Conclusions:**

Visceral osteopathic treatment of the abdomen did not accelerate meconium excretion in VLBW (very low birth weight)-infants. However infants in the osteopathic group had a longer time to full enteral feedings and a longer hospital stay, which could represent adverse effects. Based on our trial results, we cannot recommend visceral osteopathic techniques in VLBW-infants.

**Trial registration:**

Clinical trials.gov: NCT02140710

## Introduction

Timing of the first and last meconium stool is critical for oral feeding tolerance and proper gastrointestinal function [1]. The time until premature infants pass their first meconium ranges from 1 hour to 27 days (median: 43 hours) [2, 3] Obstruction of the gastrointestinal tract by tenacious, sticky meconium frequently leads to gastric residuals, a distended abdomen and delayed food passage. Recent data support the concept that complete rapid evacuation of meconium plays a key role in feeding tolerance [4]. If the duration to full enteral feedings is extended, the probability to acquire infections due to intravenous access for parenteral nutrition increases and the hospital stay is prolonged.

Recently two prospective randomized trials examined whether different pharmacological interventions may accelerate delayed meconium evacuation in preterm infants [5, 6]. Neither of the applied drugs appeared to be beneficial. Thus, we were looking for an alternative, non-invasive strategy.

Osteopathic treatment with the emphasis on the relationship of the structural and functional integrity of the body and with its variety of therapeutic manual techniques seemed to be a promising approach. Osteopathic techniques might be helpful in young children with constipation [7]. As involvement of the vagus nerve can contribute to autonomic imbalance, osteopathic treatment should focus on the atlanto- axial and atlanto-occipital joint. Both the pelvic and abdominal diaphragms should be examined and treated since ptosis of the viscera may be a problem in constipation. Treating the child with fascial release of the colon can be extremely helpful [7]. In children with cerebral palsy osteopathic treatment with fascial release, iliopsoas muscle release, sphincter release, and bowel mobilizations was successful to alleviate chronic constipation [8]. The pathophysiology of constipation is closely related to delayed meconium excretion. Treating the abdomen of premature infants with visceral osteopathic techniques might mobilize meconium mechanically from the small intestines and proximal colon. Therefore we hypothesized that repeated visceral osteopathic treatment during the first week of life accelerates meconium evacuation in premature infants, and thereby enhances feeding tolerance in this population.

## Methods

### Design

The study design was performed as a prospective, randomized, controlled trial at the NICU (Neonatal Intensive Care Unit), Department of Pediatrics, Medical University of Vienna/Austria. Patient recruitment started at first December 2010 and lasted until 28^th^ of February 2012. Follow up was completed on 31^st^ of May 2012. Written informed consent was obtained from the parents after full explanation of the procedure. Infants with a birthweight <1500g and a GA <32 weeks were included, stratified according to their GA (gestational age; < 28 vs. ≥ 28 weeks) and assigned randomly to the intervention or control group. Exclusion criteria were major congenital malformations and known gastrointestinal abnormalities. The trial was approved by the Ethics Committee of the Medical University of Vienna (EK: 126/2010; approved at 12.5.2010). The trial was registered at Clinical trials.gov: NCT02140710 subsequently after finishing the study. The parents of the individual shown in the manuscript (striking image) and video (S1 Film) have given written informed consent (as outlined in PLOS consent form) to publish this case details. The study was planned at the end of 2009- at that time the Medical University of Vienna recommended to register randomized controlled trials on the upcoming new trial registration Clinical trial.gov, especially when drugs or medical products were investigated. The present study reports on methods of alternative medicine and in 2009/2010 it was not common to register these type of studies in one of the corresponding networks. However we decided to register the study after completing. The authors confirm that all ongoing and related trials for this drug/intervention are registered.

### Randomisation

Based on the order in which they enter the study, infants were assigned a randomisation number. These randomisation numbers have been linked to one of the study groups, using SAS software (SAS Institute Inc.100 SAS Campus Drive Cary, NC 27513–2414, USA). The details of the randomisation are known to the investigator and to the site staff. The infant received the treatment that corresponded with his/her randomisation number

### Study groups

Infants in the intervention group received an osteopathic treatment algorithm within their first 48 hours of life according the following protocol adapted from visceral treatment of adults by Barral and Finet [9]:

- Global and local listening and local on the abdomen:

The infant was always positioned in the supine position. Turning and rolling the baby during osteopathic treatment would be exhausting for the infant- therefore it was avoided. The touch and focus was on the fascial tension of the abdomen- the practitioner was looking for dysfunction with two or three fingers on the abdominal fascia moving the fascia toward the place of greatest tension.

- Release lower ribs and thoracic diaphragm:

The diaphragm is the propelling force for the movement of the colic flexures and treatment of the diaphragm has also an impact on circulatory relationships [9]- therefore we included this technique in the treatment algorithm. Furthermore the respiratory movement of the 12th rib is essential for the rhythmic flow of lymph in the thoracic duct where it enters the thorax under the median accurate ligament. Thoracic respiration is responsible for 50% of the lymph movement in the thorax and diaphragmatic movements especially are essential for enabling the lacunae on the undersurface to absorb the abdominal fluid, the gut being the largest lymphoid organ in the body [10]. The practitioner spread two fingers grasping the lower rib cage and applied mobilizing pressure to the thorax in translation, altering rhythmically to the left and to the right.

- Pylorusrelaxation

To find the pylorus, the practitioner looked for its approximate projection on the stomach wall. For this purpose, the practitioner had to move from the navel about one finger width cranially. From there fingers were placed slightly next to the right, next to the median line. At this point, fingers slowly slid posteriorly into the abdomen. Once palpation advanced deeply enough, a lens-sized solidification could be palpated. Small circulations, vibrations or inhibitions on this point were applied until the tonus and sensitivity were clearly reduced. Only slight pressure was applied until allowing the superficial structures to relax.

- Release of the Duodenum and the C-Loop

In the present study infants were always treated in the supine position. Both techniques described above were combined together. The fascial structures of the duodenum were mobilized with the index finger along the anatomical structure with a slight pull in the of direction functional movement from oral to aboral. The pull was intensified during inspiration and maintained during expiration.

- Small intestine diagnosis- Lifting the gut and bringing it to a stillpoint

The technique was applied with a two- finger (thumb and index finger) pinch grip grasping the whole small intestine and was combined with the root of mesentery diagnosis. The grip was applied slightly further posteriorly into the abdomen, to capture a part of the small intestinal loops as well. The anterior pull could thus also include the root of mesentery. This technique is very effective for adhesions/fixations but must be applied with caution because it can be very painful. Small clockwise movements were applied and synchronized with in and exhalation until tissue relaxed. Finally the gut was lifted and brought to stillpoint.

- Mobilisation of the ileocoecal valve

The technique was applied with the index fingers of both hands. A line was drawn from the right anterosuperior iliac spine to the navel and divided into two halves. Both fingers slid slowly posteriorly to the abdomen- the finger closer to the midline fixed the ileocoecal valve and the finger of the other hand slightly pulled to lateral in direction of the anterosuperior spine. Small vibrations or inhibitions were applied until the tissues relaxed.

- Mobilisation of colon ascendens, transversum, descendens with treatment of the Toldt fascia

The individual parts of treatment were all taken together in a fluently applied deep pull, starting caudally in the ascending area, rotating clockwise to the transversum area and going down caudally to the Colon descendens. Finally the Toldt fascia was mobilized with a pinch grip of the thumb, which was positioned on the ascending/descending colon and the index finger, which was positioned posterior. The fascia was mobilized with rebounds and frictions.

- Root of sigmoid diagnosis and manipulation [7]

The root of sigmoid is a thickening of the peritoneum, extending from the sigmoid to the area of the bifurcation of the iliac vessels. The mechanical tension extends further to the area of duodeno-jejunal junction. The technique was applied with one or two fingers (thumb and index finger) pinch grip grasping the mesocolon. Small clockwise movements were applied until tissue relaxed.

- As the 10th cranial nerve influences the intestines’ function by relaxing the sphincters and thus increases gut motility, treatment of the parasympatic nerval system should be always in involved. The vagus nerve was treated by craniosacral therapy via the sacrum. The hand of the osteopath was put under the sacrum of the infant and cranial treatment was given as long as the infant’s body reached a stillpoint.

The treatment algorithm shown in the attached video (S1 Film) was repeated three times during one treatment and on three days during the first week of life. The preterm infant was treated in the incubator, lying on his/her back. All infants received non invasive respiratory support by continuous positive airway pressure (Infant flow Eumedics, Austria). The Infant flow device is fixed on the head of the infant with a cap- therefore any touch or therapy via the head was not accomplishable. Heart rate, temperature, respiration rate and oxygen saturation of the infant were monitored during the whole procedure. Treatment was scheduled approximately one hour after feeding to avoid a hungry baby on the one hand and unpleasant pressure on the full stomach on the other hand. If a second person was available (student), all treatments were performed under facilated tucking. To avoid irritation of the immature skin the abdomen of the infant was lubricated with an ointment. If the infant showed any signs of discomfort or cardiovascular instability, the treatment was withheld until symptoms or conditions disappeared. The control group did not receive any intervention.

### Definition of primary outcome

Primary outcome parameter was specified as complete meconium excretion. The time to complete meconium evacuation was defined as day of life on which the last meconium was passed. The nursing staff assessed the quality of stools as “meconium” (black, thick, sticky) or “non meconium” by appearance and documented data into the electronic patient documentation system. Documentation of stool consistency, colour and amount was continued until the end of the infants’ stay at the NICU. Secondary outcome was defined as the introduction of enteral feeding in days, feeding volume on day 14^th i^, time to full enteral feeding in days and hospital stay. The observation period was censored when the infant was transferred or discharged.

### Standardized feeding regimen

All preterm infants routinely received a gastric tube during the first hour of life. Within the first 12 hours of life minimal enteral nutrition was started, defined as 1ml of nutrition (preterm formula or breast milk) every 3 hours [11]. If no breast milk was available, undiluted hydrolysed preterm formula (Prematil HA/Milupa, Puch, Hallein Austria) or Beba F/Nestle Vevey, Switzerland) was used [12]. The colour and amount of gastric residuals was assessed before each feeding by aspiration via a gastric tube. Colour and consistency of gastric residuals were assessed as clear (mucous), milk-coloured, clear green, green with flakes, blood-tinged or haemorrhagic. The sole presence of clinical conditions such as infection, hypotension or respiratory support and the subjective impressions of the attending nursing staff did not influence the feeding strategy. The following were considered as signs of feeding intolerance was defined according [2, 13]: (i) increase in abdominal girth of >2 cm compare to the previous measurement; (ii) guaiac-positive stools; (iii) a single gastric residual greater than 3mL/kg body weight; (iv) grossly bloody stools; (v) emesis; (vi) ileus; (vii) bile-stained gastric residuals; (viii) radiologic evidence of necrotizing enterocolitis (NEC) (pneumatosis or portal venous gas). If signs of feeding intolerance occurred all stools were tested for presence of haemoglobin (Haemoccult; Beckman-Coulter, Krefeld-Fischlen, Germany). Mild feeding intolerance was defined when one of the symptoms (i), (ii) or (iii) occurred and feeding was discontinued for six hours. Severe feeding intolerance was defined if one of these signs was accompanied by symptoms of illness such as sepsis, or if two signs occurred together, or if symptoms (iv) through (vii) occurred. Feeding was withheld according to the clinical condition of the infant, for six hours after extubation and during indomethacin therapy.

The daily amount of nutrition was increased by 20ml/kg/d [14]. Full enteral feedings were defined as 140ml/kg [15]. At an enteral intake of 100ml/kg, breast milk was supplemented with breast milk fortifier (BMF), e.g.: Aptamil FMS/Milupa (Puch, Hallein Austria), FM 85/Nestle (Vevey, Switzerland).

### Data collection

All infants were treated according the same standard care procedures for VLBW-infants used at our department. Infants were monitored documenting the clinical condition of the abdomen (size, tension, peristaltic, apparent standing intestinal loops), stooling pattern, ventilation and ventilator support (positive end expiratory pressure-PEEP) every hour, during the first 48 hours after osteopathic treatment. Blood pressure was monitored continuously by an arterial line during the first three days of life. During the further study period the following parameters were recorded daily: body weight, volume of enteral and parenteral fluids, volume and colour of gastric residuals before every meal, abdominal girth, presence of gross abdominal distension, presence of persistent visible loops without peristalsis, presence of abdominal tenderness, stool pattern, and respiratory support. Growth restriction was defined as birthweight below the 10% percentile measured with the Fenton growth charts. Concomitant application of suppositories and enemas were recorded as well as laboratory parameters of infection (full blood count, C reactive protein, interleukin-8, blood culture) and antibiotic therapy. Necrotising enterocolitis (NEC) was defined according to the stages by Bell as proven NEC grade 2a [16].

### Statistical analysis

Based on a previous study investigating meconium passage in VLBW infants [5], a sample size estimation [17] indicated that a total of 40 infants would suffice to detect a 20% difference in the outcome between the groups with ß- error of 0.08 and a significance level of 0.05. Data were checked for normal distribution visually by histograms and with the Kolmogorov-Smirnow test. Data are expressed as mean 95% confidence intervals or median and the range as appropriate. As several data set were non-normally distributed, all comparisons were performed using non-parametric tests to increase robustness. The “Chi2- test” and Fisher’s exact test were used for dichotomous (demographic) variables. For all tests, a p-value < 0.05 was considered to indicate statistical significance. SPSS statistical software system (SPSS Inc., Chicago, IL, version 10.0) was used for all calculations.

## Results

### Study population

**Fig 1:**
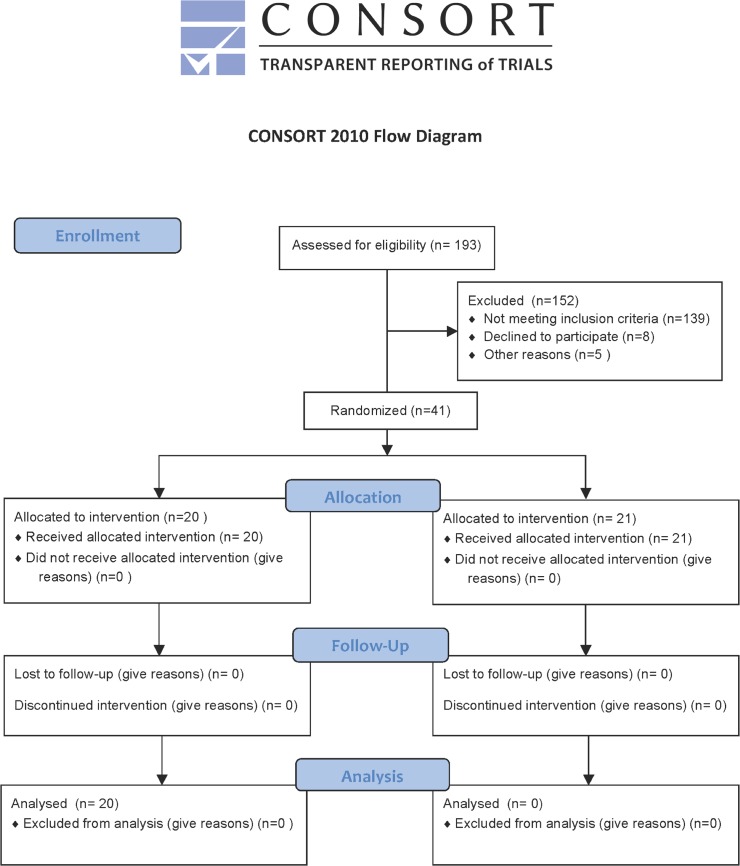
Consort form.

During a 14-month trial period from December 2010 to February 2012 193 infants were eligible for enrolment in the study. Infants were excluded for the following reasons: informed consent not obtained in time (n = 139), parental refusal (n = 8) and 5 infants died before randomization. Therefore the final cohort included 41 infants (Fig 1). Baseline characteristics between study groups were balanced and are summarized in Table 1. No differences between the groups were observed. There were no significant differences in baseline characteristics between groups (p>0.05).

**Table 1:** Demographic characteristics of the study population (Mann- Whitney- U test).

	Control group (N = 20)	Intervention group (N = 21)	p-value
	Median (Range)	Median (Range)	
Birthweight (grams)	765 (503–1150)	730 (380–1400)	0.76
Length at birth (cm)	35.5 (30.5–40)	33 (27.5–40)	0.25
Head circumference at birth (cm)	24.5 (21–27)	23 (20–28.5)	0.66
Gestational age at birth (days)	193 (167–204)	182 (163–218)	0.74
Gestational age at birth (weeks)	28+2 (23+6–30+0)	26+1 (23+2–31+1)	0.74

Outcome data on relevant neonatal morbidities were also distributed equally and are given in Table 2. These data indicate that visceral osteopathic treatment had no negative effect on major outcome of our patients. Especially the incidence of NEC remained stable in the intervention group and showed no difference to controls.

**Table 2:** Morbidity and mortality of the study population (chi2- test).

	Control group (N = 20)	Intervention group (N = 21)	p-value
	N (%)	N (%)	
Male sex	6 (30)	6 (28.6)	0.92
Deceased	0 (0)	1 (4.8)	0.29
SGA	6 (30)	6 (28.6)	0.92
NEC	1 (5)	2 (9.5)	0.52
NEC surgery	0 (0)	2 (9.5)	0.14
IVH I+II	5 (25)	5 (23.8)	0.93
IVH III+IV	1 (5)	1 (4.8)	0.97
PDA	14 (70)	15 (71.4)	0.56

(SGA = Small for gestational age, NEC = Necrotizing enterocolitis, IVH = intraventricular haemorrhage, PDA = persisting ductus arteriosus) There were no significant differences in outcome data between groups (p>0.05).

### Primary and secondary outcome

Clinical characteristics including feeding and stooling variables of study patients are given in Table 3. In the intervention group the primary endpoint meconium evacuation lasted median 7.5 days (95% Cl: 6.4–9.4 days) and 6 days (95% CI 5.2–9.1 days; n.s.) in the control group. A post-hoc subgroup analysis showed no difference in meconium evacuation between infants with a birthweight below 1000g and 1001-1500g. In a correlation analysis we tested for correlation between time for complete meconium evacuation and birthweight or gestational age. In the control group higher birthweight was associated with later meconium evacuation (r = -0.56, p = 0.09). This association was not found in the intervention group. Gestational age wasn’t a predictor in either group or both groups together.

**Table 3:** Primary and secondary outcome.

	Control group (N = 20)	Intervention group (N = 21)	p-value
	Median (Range)	Median (Range)	
First meconium (days)	2 (1–7)	2 (1–5)	0.16
Last meconium (days)	6 (2–21)	7.5 (3–18)	0.11
Feeding amount on 14^th^ day of life (ml/kg)	84 (11–158)	99 (4–133)	0.74
Full enteral feedings (day of life)	26 (11–52)	34 (14–99)	**0.02**
Duration of stay in the NICU (days)	66 (13–139)	100 (13–229)	0.14
Weight at discharge home (gram)	2920 (2060–5428)	3255 (1948–6225)	0.58

Clinical characteristics of the study population including feeding and stooling pattern (Mann-Whitney-U test)

(NICU = Neonatal Intensive Care Unit).

Time to full enteral feedings was 8 days longer in the intervention group (median 34 days, 95% Cl: 30–48 days) than in the control group (median 26 days, 95% Cl: 20–31 days; p = 0.02), which was significant. This was associated with a 34 days longer stay in the NICU in the intervention group than in the control group (Table 3). In both groups enteral nutrition started on the second day of life (= median; Intervention: 95%CI: 1.2–2.8 day; controls 95% CI: 1.6–2.5day; n.s.). All infants in the study received respiratory support by continuous positive airway pressure (C-PAP; Table 4). Infants were on c-PAP for 32 days (= median, 95%CI: 20–51 days) in the intervention group and for 20 days (= median, 95% CI: 16–32 days) in the control group, respectively. Thirteen infants in the intervention and 7 infants in control group needed mechanical ventilation. Mean days on respirator were 12 days (95% CI: 4–21 days) in the intervention group and 4 days (95%CI: 0–6 days) in the control group (p = 0.09). Overall infants in the intervention group were 3-times longer on ventilator than in the control group. Although this achieved no statistical significance there was a trend that the infants in the intervention group were the smaller and sicker ones.

**Table 4:** Data on respiratory support.

	Control group (N = 20)	Intervention group (N = 21)	p-value
	N (%)	N (%)	
Respiratory support C-PAP	20 (100)	21 (100)	1
Respiratory support mechanical ventilation	7 (35)	13 (61.9)	0.085
	Median (Range)	Median (Range)	
Days on C-PAP	19,5 (2–57)	32 (3–150)	0.40
Days on mechanical ventilation	0 (0–15)	5 (0–75)	0.09

### Suppositories and enemas

As suppositories or enemas might have an impact on meconium evacuation we analysed the frequency how often these therapies were administered. No differences were observed between groups in terms of receiving glycerine suppositories or enemas until complete meconium excretion was achieved (Table 5).

**Table 5:** Enemas or suppositories applied in the study (Fisher exact-test).

	Control group (N = 20)	Intervention group (N = 21)	p-value
	N (%)	N (%)	
Glycerine suppositories once until complete meconium evacuation	4 (20)	7 (35)	0.27
Glycerine suppositories multiple until complete meconium evacuation	13 (65)	11 (52.4)	0.31
Enema once until complete meconium evacuation	5 (25)	2 (9.5)	0.18
Enema multiple until complete meconium evacuation	17 (85)	18 (85.7)	0.64

### Tolerance of the osteopathic procedure

In general the procedure was well tolerated. All infants were monitored during the treatment and no one showed signs of cardiorespiratory instability, apnoea or pain. Only 1 infant (4.8%) reacted with agitation and showed signs of discomfort- after a short break of five minutes the patient calmed down and the treatment was continued without further problems.

## Discussion

This prospective, randomized, controlled trial examined the effect of visceral osteopathic treatment on meconium evacuation in VLBW-infants. Osteopathic treatment was well tolerated without any impact on respiratory or cardiovascular system. However, visceral osteopathic treatment did not accelerate complete meconium excretion. Time to full enteral feedings was significantly longer in the osteopathic group as compared to controls, which can be interpreted as adverse effect.

Osteopathy has been considered a form of complementary medicine, emphasizing a holistic approach and the skilled use of a range of manual treatment [18]. The Osteopathic Terminology defines somatic dysfunction as “impaired or altered function of related components of the somatic system: skeletal, arthrodial, and myofascial structures, and related vascular, lymphatic and neuronal elements [7]. In the present study delayed meconium excretion of the preterm infant was defined as the somatic dysfunction to be treated. In this special group of patients proper function of the organ has never been established and the somatic dysfunction is determined by the immaturity of the intestinal motor mechanisms and associated feeding problems [19]. So far, osteopathic treatment is a rarely applied method to treat pathologic conditions in premature infants and has already started to explore the field of neonatal intensive care. Unfortunately, there is a lack of good research evidence to support many of the claims and assertions made by osteopathy. Although osteopathic treatment is non- invasive it might have an impact on the organism and it is important to prove efficiency and efficacy of the therapies.

In the present study visceral osteopathic treatment was applied in preterm infants with a median birthweight between 700-800g and a gestational age about 28 weeks. Our smallest patient had a birthweight of 422g and a gestational age of 23+2. This definitely represents the minimum weight and age of viability. Infants with extremely low birth weight are more susceptible to all of the possible complications associated with premature birth, both in the immediate neonatal period and after discharge from the nursery. Do these babies have any chance to survive with a proper neurodevelopmental outcome? Every day inside the mother’s womb increases the chance of survival for a micropreemie, and every week is a major significance continuing to push that percentage higher. Recently our team published data on survival and neurodevelopmental outcome in our hospital [20]. Infants born between the 23+0 and 25+6 week of gestation survived in 68.1%, Infants born between the 26+0–27+6 survived in 84.8%, respectively. 9.4% of the survivors suffered from a major cerebral lesion (intraventricular haemorrhage and periventricular leukomalacia), which is associated with a poor neurodevelopmental outcome like cerebral palsy or major loss in vision or hearing. Therefore the majority of these small micropreemies survived their stay in the NICU with almost no or only mild impairment. To the best of our knowledge no one has published data on osteopathic treatment such tiny micropreemies previously.

The question of the present study was generated out of a relevant clinical problem in neonatal intensive care medicine. Visceral osteopathic techniques are based on the specific placement of soft manual forces to encourage the normal mobility, tone and motion of the viscera and their connective tissues [21]. As gastric motility is strongly influenced by the vagal nerve the treatment should address to the vagal nerve to increase the range of motion of the several segments and release facial tensions. In a prospective randomized trial preterm infants received moderate pressure massage and were compared to control with light pressure massage [21, 22]. The moderate massage stimulated the vagal nerve leading into increased gastric motility. This turned into greater weight gain and increased the release of insulin and IGF-1 (insulin growth factor) [22]. Field et al reported a greater weight gain of 21–47% in preterm infants treated with moderate pressure massage as compared to controls. The change in insulin and IGF-1 suggested two parallel pathways via which massage therapy leads to increased weight gain: 1) insulin release via the celiac branch of the vagus and 2) increased gastric activity via the gastric branch of the vagus [22].

The pressure on the abdomen given in visceral osteopathic treatment is comparable with moderate pressure massage reported by Field [21]. Although the intention of the present study was well defined and the selected techniques were convenient to serve the purpose, the study failed in success. Meconium excretion lasted 7.5 days after visceral osteopathic treatment and 6 days without. Time to full enteral feedings lasted 8 days longer in the intervention group than in the control group, which was significant. This was associated with a 34 days longer stay in the NICU as compared to controls. Furthermore infants in the intervention group needed 12 days longer respiratory support by C-PAP and were 3-times longer on ventilator than in the control group. Although baseline and outcome data were well balanced between groups, these data indicate, that infants in the intervention group were eventually sicker. As this trial was randomized, it is possible that these results of secondary outcomes represent adverse effects of osteopathic treatment, if theses findings are not due to chance because of multiple comparisons.

So far only a few high quality randomized controlled trials focussing on the therapeutic effectiveness of osteopathic treatment in premature infants and neonates are published and most of them failed to prove efficacy: a previously published review evaluated osteopathic manipulative therapies (OMT) in paediatric conditions and identified only 1 study in preterm infants [23]. The authors of the review mainly criticized low methodical quality and paucity of the analysed osteopathic studies. More than half of the studies (9 of 17 randomized controlled trials) did not report on any statistical calculations for effect size. Five of 17 trials had a high risk of bias with regard to adequate sequence generation. Nine of 17 trials had a high risk of bias with regard to allocation concealment. Twelve of 17 trials had a high risk of bias with regard to patient blinding and nine to assessor blinding. Six trials had a high risk of bias with regard to addressing of incomplete data and selective outcome reporting and 4 trials failed to provide any details about the osteopathic manipulative therapies, making them impossible to be replicated. So overall the quality of the reported randomized controlled trials was poor and no trial was free of major methodical limitations. The evidence from randomized controlled trials of OMT for treating paediatric conditions is thus limited, weak, and contradictory.

This was the first prospective randomized controlled study on visceral osteopathic techniques applied in premature infants. The study provides a sampled size calculation and primary and secondary outcomes are clearly defined. Applied visceral osteopathic techniques and their adaptation were well described, guaranteeing reproducibility by other osteopaths.

One limitation was that in 139 cases informed consent could not be achieved in time: osteopathic treatment had to be applied during the first 48h hour of life- therefore informed consent had to be obtained during this time span which was often unfeasible. Secondly, parents had to be contacted twice: The guidelines of good clinical practice (GCP) postulate, that there has to be a timespan between information of the parents/patients and signing the informed consent- ideally”over the night”- so the parents should have the opportunity to consider participation in the study. We always encouraged the parents to take their time afterwards asked again for informed consent. Therefore it happened, that informed consent was signed after the first 48 hours of the infant’s life and the infant had to be excluded. One more limitation is the small sample size, which may have prevented the detection of more significant effect size. However, irrespective of outliers there wasn’t even a trend for an improvement in median time of meconium evacuation in the Intervention group.

Furthermore the very tiny body and the small surface of the abdomen was a challenge for the therapist to apply visceral osteopathic techniques properly. It is conceivable that the techniques were not applied precisely and therefore failed to be effective. In addition preterm infants are very susceptible to touch. Although the infants in our study showed no signs of discomfort during osteopathic treatment it is conceivable that the algorithm was too rude and therefore failed to be effective. One limitation is the small sample size which may have prevented the detection of more significant effect size.

## Conclusions

In the present trial we investigated the effect of visceral osteopathic treatment on meconium evacuation in VLBW-infants. Osteopathic treatment was well tolerated without any impact on respiratory or cardiovascular system. However, the results indicate that visceral osteopathic treatment did not accelerate complete meconium excretion. Time to full enteral feedings was significantly longer in the osteopathic group as compared to controls, which is a potential adverse effect of visceral osteopathic treatment. Based on our trial results, we cannot recommend visceral osteopathic techniques in VLBW-infants.

## Supporting Information

S1 Checklist Consort Checklist. (DOC)

S1 Film Visceral osteopathic treatment of an extreme low birth weight infant. The movie shows the osteopathic treatment algorithm applied in a premature infant with a birthweight of 523 gram and a gestational week of 24+2.For the sake of brevity the video was shortened from 24 minutes to 1 minute.
(MOV)

S1 Protocol Study proposal. (DOC)
