# Correction: Does Visceral Osteopathic Treatment Accelerate Meconium Passage in Very Low Birth Weight Infants?- A Prospective Randomized Controlled Trial

**DOI:** 10.1371/journal.pone.0187784

**Published:** 2017-11-02

**Authors:** Nadja Haiden, Birgit Pimpel, Alexandra Kreissl, Bernd Jilma, Angelika Berger

In this *PLOS ONE* article, short excerpts of text were duplicated verbatim from the sources listed below. In each case where the original source is a previously published journal article or book, the original publication was properly cited in the *PLOS ONE* article. However, the authors acknowledge that the text from these sources should have been either reworded or reproduced with quotation marks. The authors declare that the text overlap has no effect on the results and conclusions of the study and apologize for these instances of overlap.

The following sentence in the Introduction section overlaps with the text from Reference 7 in the original article:

“Both the pelvic and abdominal diaphragms should be examined and treated since ptosis of the viscera may be a problem in constipation.”

The following sentence in the Methods section overlaps with the text from Reference 10 in the original article:

“Thoracic respiration is responsible for 50% of the lymph movement in the thorax and diaphragmatic movements especially are essential for enabling the lacunae on the undersurface to absorb the abdominal fluid, the gut being the largest lymphoid organ in the body.”

The following portion in the Methods section overlaps with the text from Reference 9 in the original article:

“To find the pylorus, the practitioner looked for its approximate projection on the stomach wall. For this purpose, the practitioner had to move from the navel about one finger width cranially. From there fingers were placed slightly next to the right, next to the median line. At this point, fingers slowly slid posteriorly into the abdomen. Once palpation advanced deeply enough, a lens-sized solidification could be palpated. Small circulations, vibrations or inhibitions on this point were applied until the tonus and sensitivity were clearly reduced.”

The following sentences in the Discussion section overlap with the text from Reference 22 in the original article:

“In a prospective randomized trial preterm infants received moderate pressure massage and were compared to control with light pressure massage.”

“The moderate massage stimulated the vagal nerve leading into increased gastric motility.”
