# Supplementary material for: Does Visceral Osteopathic Treatment Accelerate Meconium Passage in Very Low Birth Weight Infants?- A Prospective Randomized Controlled Trial
Source: PLoS One. 2015 Apr 15;10(4):e0123530. doi: 10.1371/journal.pone.0123530 (PMC4398405; doi:10.1371/journal.pone.0123530)
Supplement: S1 Protocol — (DOC) [file pone.0123530.s003.doc]

**Studyproposal**

**Titel:**

**The impact of visceral osteopathic treatment on the meconium evacuation in very low birth weight infants**

**Masterthesis Osteopathy by Doz. Dr. Nadja Haiden**

**at the Donauuniversity Krems**

**Introduction:**

The physiologic immaturity of the intestinal motor mechanisms and the associated feeding problems are one of the challenges for neonatologists in the treatment of very low birth weight infants (VLBW- infants= with a birthweight <1500gram). Timing of the first and last meconium stool is critical for oral feeding tolerance and proper gastrointestinal function [1]. The vast majority of term infants (99.8%) pass their first meconium within 48 hours of life [2], while premature infants show a wide variability. The time to pass their first meconium ranges from 1 hour to 27 days (median: 43 hours) ([3, 4]. Obstruction of the gastrointestinal tract by tenacious, sticky meconium frequently leads to gastric residuals, a distended abdomen and delayed food passage. Recent data support the concept that complete rapid evacuation of meconium plays a key role in feeding tolerance [5]. Duration to full enteral feedings is extended, the probability to acquire infections due to intravenous access for parenteral nutrition increases and hospital stay of the infant prolongates. Data from a study including 21 preterm infants with the diagnosis “Meconium obstruction in the very low birth weight premature infant” showed that tenacious meconium is most frequently located in the distal ileum [6]. In that study, various kinds of enemas were demonstrated to be effective and safe for promoting the evacuation of tenacious meconium plugs during or shortly after instillation. Resolution of obstruction with glycerin suppositories, saline, acetylcystein or non-absorbable contrast media was conducted in 70% of symptomatic infants, thereby avoiding surgical removal of the plug. Recently, the study group around Haiden et al performed a prospective randomized trial; to determine whether repeated prophylactic applications of small volume glycerin enemas are effective in accelerating passage of meconium in VLBW infants [7]. The authors did not find a correlation between application of enemas and meconium evacuation. A reason for the ineffectiveness of glycerin enemas was supposed to be that the volume used was too small to mobilize tenacious meconium sufficiently from deep parts of the colon and small bowel (Colon ascendes, terminal Ileum).

**Osteopathy**

Osteopathy is a complete system of medical care with a philosophy that combines the needs of the patient with current practice of medicine, surgery and obstetrics, that emphasizes the interrelationship between structure and function and that has an appreciation of the body’s ability to heal itself[8]. Osteopathy has been considered a form of complementary medicine, emphasizing a holistic approach and the skilled use of a range of manual and physical treatment Techniques and methods which are non- invasive, and conducted by exclusively using the osteopaths hands as a diagnostic and treatment tool.

Techniques of osteopathic treatment[9]:

Cranial osteopathy: Cranial osteopathy is a set of theory and techniques that have been developed from the observations of [Dr William Sutherland](http://en.wikipedia.org/wiki/William_Garner_Sutherland) that the plates of the cranium permit microscopic movement or force dissipation and that there is a 'force' or rhythm that is operating in moving the plates of the skull.[10] Cranial osteopathy is said to be based on a *primary respiratory mechanism*, a rhythm that can be felt with a very finely developed sense of touch. Some osteopaths believe that improving dysfunctional cranial rhythmic impulses enhances cerebral spinal fluid flow to peripheral nerves, thereby enhancing metabolic outflow and nutrition inflow. It has gained particular popularity in the treatment of babies and children.

Osteopathic manipulative medicine: The goal of Osteopathic manipulative medicine is the resolution of what many osteopaths call [somatic dysfunction](http://en.wikipedia.org/wiki/Somatic_dysfunction) in an attempt to aid the body's own recuperative faculties. Osteopathic manual treatment of the musculoskeletal system employs a diverse array of techniques. These are normally employed together with dietary, [postural](http://en.wikipedia.org/wiki/Human_position), and occupational advice, as well as counseling in an attempt to help patients recover from illness and injury, in an attempt to minimize or manage pain and disease.

Visceral osteopathy: Proponents of [visceral](http://en.wikipedia.org/wiki/Visceral) osteopathy state that the visceral systems (the internal organs: digestive tract, respiratory system, etc.) rely on the interconnection [synchronicity](http://en.wikipedia.org/wiki/Synchronicity) between the motion of all the organs and structures of the body, and that at optimal health this harmonious relationship remains stable despite the body's endless varieties of motion. The idea is that both *somato-visceral* and *viscero-somatic* connections exist, and manipulation of the somatic system can affect the visceral system (and vice-versa).Practitioners contend that visceral osteopathy relieves imbalances and restrictions in the interconnections between the motion of all the organs and structures of the body—namely, nerves, blood vessels, and fascial compartments. During the 1940s, osteopaths like H V Hoover and M D Young built on the work of Andrew Taylor Still to create this method of assessment and manipulation. The efficacy and basis of this treatment remains controversial even within the osteopathic profession.

Especially in children these techniques are well accepted because they are likely to cause no pain or other negative side effects. However, no evidence based data concerning safety and efficacy of osteopathic treatment in infants- especially in premature infants- are available so far.

Therefore, in the present study visceral osteopathic techniques are applied with the aim to accelerate meconium evacuation and establish proper gastrointestinal function in VLBW-infants . Furthermore the safety and efficacy of the applied osteopathic techniques should be surveyed.

**Materials and Methods:**

**Aim of the study:**

The aim of this prospective randomized trial is to determine whether early osteopathic treatment of bowel and colon has an influence on meconium evacuation in very low birth weight infants.

**Hypothesis:**

We hypothesize that a standardized treatment algorithm of osteopathic bowel techniques accelerates complete meconium evacuation in preterm infants.

**Patients and Inclusion criteria:**

Premature infants with a birth weight ≤1500 g and a gestational age (GA) ≤32 weeks are eligible for inclusion in the study.

**Exclusion criteria:**

Exclusion criteria were major congenital malformations and known gastrointestinal abnormalities.

**Design:**

The study design is performed as a prospective randomized controlled trial at the Neonatal Intensive Care Unit (NICU), Department of Pediatrics, Medical University of Vienna/Austria. Infants with a birthweight <1500g and a GA <32 weeks will be included, stratified according to their GA ( < 28 vs. ≥ 28 weeks) and assigned randomly to the intervention or control group. Randomization assignment is performed using sealed opaque envelopes that are grouped in an even blocked size design (block size 4) by stratification variable (GA).The study will be approved to the Ethics Committee of the Medical University of Vienna. Written informed consent will be obtained from the parents after full explanation of the procedure. Infants will be treated according the same standard care procedures for VLBW-infants used at our department.

Study groups:

Infants in intervention group will receive osteopathic treatment within their first 48 hours of life according the following protocol:

All techniques are applied in the supine position:

1. Global listening- any further treatment methods employed to improve mobility must be slow and gentle
2. Release lower ribs and thoracic diaphragm
3. Small intestine diagnosis- Lifting the gut and bringing it to a stillpoint
4. Root of mesentery diagnosis (and manipulation)
5. Mobilisation of the ileocoecalic valve
6. Mobilisation of colon ascendens, transversum, descendens
7. Root of sigmoid diagnosis and manipulation
8. As the 10^th^ cranial nerve influences the intestines’ function by relaxing the sphincters and thus increases gut motility treatment of the parasypmatic nerval system should be always in involved

The treatment algorithm will be repeated three times during the first week of life.

No intervention is planned in the control group.

After admission of the patient to NICU, the infant received primary care according to

the standard principles of neonatal intensive care (monitoring of oxygen saturation,

heart rat, blood pressure, temperature, fluid intake and urine rate, intravenous access

by periphery or central venous line).

The nursing staff assesses the quality of stools as “meconium” (black, thick, sticky) or

“non meconium” by appearance and documented data into the “carevue”

documentation system.

The time to complete meconium evacuation is defined as day of life on which the last meconium was passed. Documentation of stool consistency, colour and amount will be continued until the end of the infants’ stay at the NICU. The observation period ends when the infant was transferred or discharged.

Data collection:

The following data will be recorded:

Before the first osteopathic treatment:

· Gestational age

· Birth weight

· Birth length

· Head circumference at birth

· APGAR

· Umbilical cord-PH

· Clinical condition of the abdomen (size, tension, peristaltic,

apparent standing intestinal loops)

· Stooling pattern

· Ventilation and ventilator support (PEEP)

For the whole duration of the study period, following data was recorded daily:

· Body weight

· Amount of parenteral fluids in ml/kg

· Enteral nutrition supply in ml/kg (Human milk or formula)

· Fortification of human milk

· Gastric residuals

· Stooling pattern

· Clinical condition of the abdomen (size, tension, peristaltic, apparent standing intestinal loops)

· Ventilation and ventilator support (PEEP and flow)

· Feeding intolerance

· Enemas

· Glycerin suppositories

**Relevance for the patient:**

The potential benefit for the patient consists in shortened meconium passage, improved feeding tolerance, shorter time of parenteral nutrition and intravenous access and therefore lower sepsis morbidity and shorted hospital stay.

**Adverse events and withdrawal**:

Severe adverse events leading to withdrawal from the study:

Ileus

Volvolus

NEC

death

**Statistics:**

**Sample size:**

Based on studies investigating meconium passage in VLBW infants [7], a sample size estimation [11] indicated that a total of 40 infants would suffice to detect a 20% difference in the outcome between the groups with a power of 80% and a significance level of 0.05

**Primary outcome:**

time to complete Meconium evacuation in days.

**Secondary outcome:**

introduction of enteral feeding in days, feeding volume on day 14^th^

time to full enteral feeding in days,

**Statistical analysis** :

Given the non-normal distribution of the data, all comparisons will be performed using non-parametric tests. The Mann-Whitney U test will be used to detect treatment effects between the groups. Kaplan-Meier curves will be plotted to visualize differences in the time to complete meconium evacuation between the groups. Comparison of the curves will be performed using the log rank test. Multiple Cox regression models will be used to identify the possible influence of the covariates birth weight and GA. For all tests, a p-value < 0.05 was considered to indicate statistical significance. SPSS statistical software system (SPSS Inc., Chicago, IL, version 10.0) will be used for all calculations.

**Duration of the study:**

Approximately 160-180 infants are administered to the neonatal intensive care unit/year. To recruit 40 patients it will take 4-6 months.

**LITERATURE:**
